# Confinement Effects in Well-Defined Metal–Organic Frameworks (MOFs) for Selective CO_2_ Hydrogenation: A Review

**DOI:** 10.3390/ijms24044228

**Published:** 2023-02-20

**Authors:** Xiaofei Lu, Chuqiao Song, Xingyu Qi, Duanxing Li, Lili Lin

**Affiliations:** 1 Institute of Industrial Catalysis, State Key Laboratory of Green Chemistry Synthesis Technology, College of Chemical Engineering, Zhejiang University of Technology, Hangzhou 310014, China; 2 Department of Chemical System Engineering, School of Engineering, The University of Tokyo, Tokyo 113-8656, Japan

**Keywords:** metal–organic frameworks, MOF-derived materials, confinement effects, selective CO_2_ hydrogenation, immobilization, encapsulation, size effect, synergy effect, interface catalysis

## Abstract

Decarbonization has become an urgent affair to restrain global warming. CO_2_ hydrogenation coupled with H_2_ derived from water electrolysis is considered a promising route to mitigate the negative impact of carbon emission and also promote the application of hydrogen. It is of great significance to develop catalysts with excellent performance and large-scale implementation. In the past decades, metal–organic frameworks (MOFs) have been widely involved in the rational design of catalysts for CO_2_ hydrogenation due to their high surface areas, tunable porosities, well-ordered pore structures, and diversities in metals and functional groups. Confinement effects in MOFs or MOF-derived materials have been reported to promote the stability of CO_2_ hydrogenation catalysts, such as molecular complexes of immobilization effect, active sites in size effect, stabilization in the encapsulation effect, and electron transfer and interfacial catalysis in the synergistic effect. This review attempts to summarize the progress of MOF-based CO_2_ hydrogenation catalysts up to now, and demonstrate the synthetic strategies, unique features, and enhancement mechanisms compared with traditionally supported catalysts. Great emphasis will be placed on various confinement effects in CO_2_ hydrogenation. The challenges and opportunities in precise design, synthesis, and applications of MOF-confined catalysis for CO_2_ hydrogenation are also summarized.

## 1. Introduction

Valorization of CO_2_ is a prerequisite for achieving a “carbon-neutral society” as declared in the 2015 Paris Agreement. The chemical conversion of CO_2_ into value-added and energy-intensive chemicals is a promising approach to achieve this goal, especially when driven with the green hydrogen from water electrolysis powered by renewable electricity. Accordingly, intensive research efforts have been dedicated to selective CO_2_ hydrogenation to CO, formic acid, hydrocarbons, or oxygenates [1,2,3,4,5]. However, these processes suffer from slow kinetics and low selectivity due to the strong strength of the C=O double bond (806 kJ mol^−1^) [6]. Many advances have been made in various heterogeneous CO_2_ hydrogenation catalysts, including oxide (e.g., Cu/ZnO/Al_2_O_3_ and ZnO-ZrO_2_), oxide/zeolite (e.g., Na–Fe_3_O_4_/HZSM-5 and ZnZrO_x_/SAPO-34), supported monometallic or bimetallic catalysts (Ru, Ir, Pt, Rh, PtCo, and PdRh), and immobilized molecular complexes [7,8,9,10,11,12,13,14,15,16,17,18,19,20,21,22,23]. The active sites are carefully designed to process special structural and electronic properties for desired products. For example, the inverse ZrO_2_/Cu was designed to maximize the Cu-ZrO_2_ interface, which promoted CH_3_OH synthesis activity and selectivity of supported Cu [24]. The 8 nmsized Ni supported on CeO_2_ exhibited superior CH_4_ selectivity over the 4 nm and 2 nm counterparts in CO_2_ hydrogenation due to the structure and size dependency [25]. The artificial (C_2+_) hydrocarbons or their oxygenated derivatives could be produced via the rational design of catalysts using zeolite [26,27]. Hence, the rational design of efficient catalysts is essential for achieving high catalytic activity and selectivity during CO_2_ hydrogenation [28,29].

Besides the rational design of efficient catalysts, the confinement effects are also widely utilized to stabilize active sites during catalytic CO_2_ hydrogenation [21,30,31,32]. The strong interactions between metal nanoparticles (NPs) and supports (e.g., Cu/β-Mo_2_C, Ir/CeO_2_, Ni/γ-Mo_2_N) are confirmed as an effective confinement strategy to prevent the aggregation of NPs [33,34,35]. It modifies the electronic and geometric structures of catalytic active sites. However, the catalyst structure derived from the strong metal–support interaction (SMSI) effect is limited to certain metal and support combinations. Encapsulating the NPs with porous materials or membranes (e.g., zeolite), and thus increasing their resistance to sintering, is another effective and versatile strategy [36,37,38,39]. The fixation of metal NPs with diameters of 0.8–3.6 nm into zeolite crystals (NPs@zeolite) was demonstrated to be sinter-resistant at 600–700 °C and outperformed conventionally supported metal catalysts (NPs/zeolite) during CO_2_ hydrogenation [36,37,40]. Unlike the utilization of SMSI or the encapsulation by the zeolite (with fixed tetrahedral Si/Al coordination and pore sizes <1 nm), the ordered three-dimensional metal–organic frameworks (MOFs), with uniform cavities, tunable local environments, tailorable composites, and versatility to introduce functional coordinators/groups, are highly attractive for the rational design of heterogeneous catalysis with confinement effects.

The last decades have witnessed intensive research efforts in the reticular design of various MOFs (>100,000), which have been explored in gas storage and separation, vapor sorption, catalysis, biomedical application, and chemical sensing [41,42]. This review only focuses on CO_2_ hydrogenation, where the high surface area and porosity of MOFs are composed of various organic linkers, which can strongly bind metal atoms/clusters to serve as active sites, proffering the ability to adjust the structural and electronic modularity [5,43,44]. These composite materials can combine both the confinement effect of the pores and the SMSI or synergistic effects between active sites and organic chelates/metal-oxo clusters, providing new opportunities in finely tuning the performance of CO_2_ hydrogenation [45,46,47]. Tsung and coworkers successfully encapsulated atomic sites or ultra-small NPs in MOFs via a post-synthesis method, which exhibited good activity and selectivity for CO_2_ hydrogenation to formic acid, CH_3_OH, and C_2_H_5_OH, respectively [48,49,50,51,52]. Moreover, metal or metal oxide NPs embedded in a porous carbon matrix can be produced by the pyrolysis of MOFs. Since various elements in MOFs are initially well ordered, the new structures can also contain highly dispersed single atoms, metal NPs, alloys, or dopants (e.g., N, P, S, or B) (in the case of dopant-containing organic linkers) [53,54,55]. Compared with conventional catalysts, the metal species are partially encapsulated by the deposited carbon, which exhibits enhanced thermal stability in high-temperature CO_2_ hydrogenation reaction conditions. For instance, iron (Fe)- and cobalt (Co)-containing nanomaterials derived from MOFs showed superior selectivity and stability for CO_2_ hydrogenation compared with traditional metal catalysts [56,57,58]. These pieces of evidence indicate that the introduction of well-defined MOFs or MOF-derived materials with identical coordinated environments is an effective strategy to unravel the key active sites for CO_2_ hydrogenation catalysts, and then enhance the catalytic activity and selectivity.

**Figure 1:**
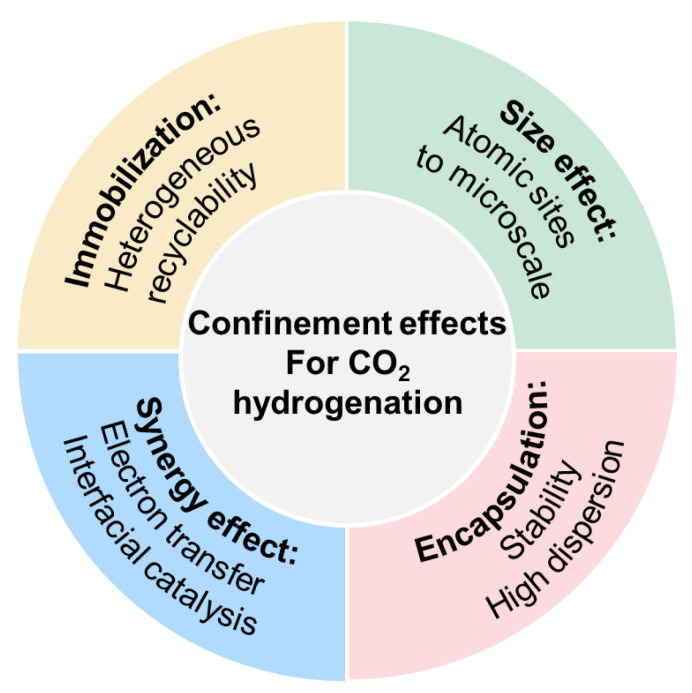
The summary of confinement effects for CO_2_ hydrogenation of MOFs or MOF-derived materials.

In recent years, several reviews have introduced advances in the synthesis, characterization, and application of zeolite-fixed metal NPs as metal@zeolite catalysts [8,37,39,59]. For example, Xiao and coworkers reviewed the reported strategies for the construction of metal@zeolite hybrid materials and described how the zeolite micropore and metal nanoparticle synergistically worked to improve the catalytic performance [37,39]. Janiak and coworkers highlighted the confinement of noble nanometals in a zeolite matrix in heterogeneous catalysis [59]. Li et al. summarized the development of MOFs encapsulating active nanoparticles as emerging composites for various catalysis, including thermal catalysis, electrocatalysis, and photocatalysis [60,61]. These works all produced excellent reviews on the preparation of porous materials–confined metals and their applications in heterogeneous catalysis. However, a comprehensive review on confined catalysis in MOFs or MOF-derived materials for CO_2_ hydrogenation is still lacking. The current review mainly focuses on the confinement effects in MOFs or MOF-derived materials during CO_2_ hydrogenation, as shown in Figure 1. Beginning with the introduction of the preparation and application of MOF-confined catalysts, the confinement effects on CO_2_ hydrogenation will be discussed including molecular complexes in the immobilization effect, atomic sites in the size effect, cage stabilization in the encapsulation effect, and adsorption and activation in the synergy effect. Furthermore, the product distribution perturbed by MOF-derived materials during CO_2_ hydrogenation will be interpreted. Finally, an outlook on the future development of MOF-based confined catalysis for CO_2_ hydrogenation will be given. We hope that the mechanistic insights interpreted by confinement effects in MOFs or MOF-derived catalysts for CO_2_ hydrogenation could facilitate the development of clear structure–activity relationships for rational catalyst design.

## 2. Confined Synthesis and Confinement Effects of MOFs as Host Materials

MOFs represent a frontier in advanced host materials for CO_2_ hydrogenation due to their tunable properties including: (1) semi-rigid and highly porous structures, (2) high physical and chemical host stability, (3) strong interaction for the stable dispersion of active species, and (4) solid Lewis or Brønsted acid characteristic. These properties provide an opportunity to synthesize stable and active catalysts using MOFs as host materials in CO_2_ hydrogenation. In this section, various synthetic strategies for MOF-confined molecular complexes, single atoms, NPs, or even micro-scale oxides were systematically summarized, including a one-pot synthetic approach (active sites confined in MOF cavities or channels), post-synthetic modification (active sites interacting with functional cavities or channels), and a two-step synthetic approach (active species surrounded by MOF membranes). For further details regarding the synthesis and characterization of various MOFs, the readers are referred to previous reviews and literature cited herein [62,63]. Moreover, the corresponding catalytic performance affected by confinement effects are detailed, indicating that rational designs of the structures of MOFs and active sites are essential in the valorization of CO_2_. 

### 2.1. Molecular Complex Encapsulation in MOFs

Compared with heterogeneous CO_2_ hydrogenation conducted at higher temperature and pressure conditions (e.g., >200 °C and >3 MPa), homogenous molecular complexes can work at mild conditions (e.g., <150 °C) [64]. The well-defined structures of molecular complexes facilitated the interpretation of reaction mechanisms at the molecular level, which promotes the optimization of catalyst design. A remaining challenge for applying molecular complexes is their immobilization to fabricate stable and recyclable catalysts for practical applications. The porosity and highly ordered structure of MOFs enable their potential as ideal host materials to improve the accessibility of catalytic sites, which also makes molecular complexes recyclable and productive. 

**Figure 2:**
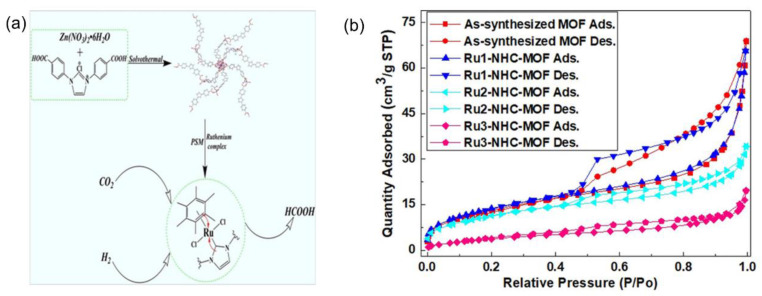
(**a**) Ruthenium complexes confined in an azolium-based MOF for selective CO_2_ hydrogenation to formic acid. Reproduced with permission. (**b**) N_2_ adsorption–desorption isotherms of as-synthesized MOF, Ru_1_-NHC-MOF, Ru_2_-NHC-MOF, and Ru_3_-NHC-MOF catalysts. Reproduced with permission [73]. Copyright 2018, Wiley-VCH.

The pioneering studies by Yoshio and coworkers demonstrated that homogeneous ruthenium–phosphine catalysts could selectively catalyze CO_2_ hydrogenation to formic acid at low temperatures [65]. Moreover, homogeneous molecular complexes containing n-heterocyclic carbenes (NHCs) or phosphorus–nitrogen–phosphorus (PNP) pincer-type ligands could facilitate CO_2_ hydrogenation due to the strong electron-donating ability, where the Ru PNP-pincer catalyst could deliver a turnover frequency (TOF) value of 1,100,000 h^−1^ at 65 °C and 4 MPa (H_2_/CO_2_ (*v*/*v*) = 3:1) [66,67,68,69,70]. However, the challenges including catalysts and products separation and recyclability hindered their industrial applications. Accordingly, the heterogenization of molecular complexes is an effective pathway, which could integrate the distinctive activity of homogeneous catalysts with the advantages of heterogeneous catalysts [71,72]. MOFs are the potential candidates to host molecular complexes. For example, Wu et al. reported various heterogeneous Ru-based molecular complexes immobilized on an azolium-based MOF via post-synthetic metalation which were examined for CO_2_ hydrogenation toward formic acid, as shown in Figure 2. The examined Ru molecular complexes included RuCl_3_, [RuCp*Cl_2_]_2_ (Cp* = pentamethylcyclopentadienyl), and [Ru(C_6_Me_6_)Cl_2_]_2_ (C_6_Me_6_ = hexamethylbenzene); the corresponding hybrid catalysts were named Ru_x_-NHC-MOF (x = 1, 2, 3). The surface areas and total pore volumes of the three as-prepared catalysts decreased due to the occupancy in the MOF pores (Figure 2b). The as-obtained Ru_3_-NHC-MOF catalyst exhibited the highest activity due to the stronger electron-donating ability of the C_6_Me_6_ ligand in the [Ru(C_6_Me_6_)Cl_2_]_2_ complex. Specifically, values of the turnover number (TON) up to 3803 were obtained at 120 °C under a total pressure of 8 MPa (H_2_/CO_2_ (*v*/*v*) = 1) for 2 h in the presence of K_2_CO_3_ as the base in N, N-Dimethylformamide (DMF) solvent. The strong polarity of the DMF solvent also facilitated the insertion of CO_2_ into the Ru−H bond, which is the rate-determining step (RDS) for CO_2_ hydrogenation [73].

**Figure 3:**
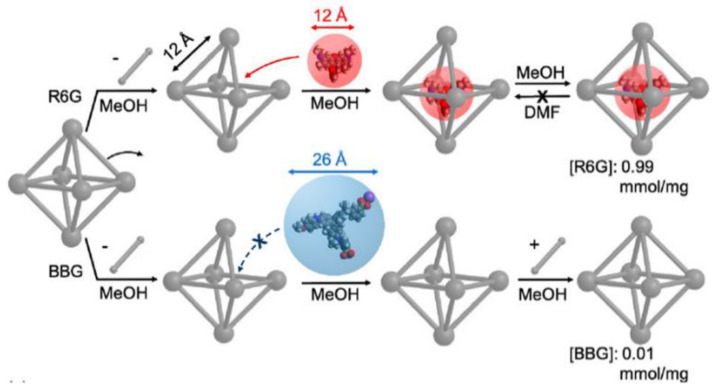
Encapsulation of molecular complexes (R6G or BBG) in UiO-66 in methanol and DMF at 55 °C for 5 days. Reproduced with permission [48]. Copyright 2018, American Chemical Society.

Host (MOF)-guest (molecular complexes) composites have already demonstrated their advantages in CO_2_ hydrogenation. However, the successful synthesis of host–guest composites is limited, and they can only be prepared via de novo synthesis and post-synthetic modification [49,74,75], where the size of molecular complexes should be smaller than the pore of MOFs. Recently, the Tsung group developed a post-synthetic approach to synthesize host–guest composites, where guest molecular complexes were larger (e.g., 3–4 times) than the aperture size of the MOF host but could also be encapsulated into MOFs via aperture-opening events [50]. Guest molecular complexes were encapsulated effectively due to the short-lived “open” states of the pores formed upon linker dissociation, which circumvented the disadvantages of previous strategies including tedious synthetic processes and poor encapsulation efficiency. The aperture-opening process occurred even in the robust MOF, which was significantly affected by the identity of the selected solvent, as shown in Figure 3. A Zr-based MOF consisting of a cubic framework of cationic Zr_6_O_4_(OH)_4_ nodes and 1,4-benzenedicarboxylate linkers (BDC) was selected as the host material, named UiO-66. The (tBuPNP)Ru(CO)HCl (tBuPNP = 2,6-bis((di-tert-butyl-phosphino)methyl)pyridine) was successfully encapsulated into UiO-66 (named [Ru]@UiO-66) by exposing UiO-66 to methanol solvent containing (tBuPNP)Ru(CO)HCl at 55 °C for 5 days in Figure 3. [Ru]@UiO-66 exhibited a comparable TON of ca. 280,000 with that of homogeneous molecular catalysts for CO_2_ hydrogenation to formic acid in DMF at 27 °C and 1.5 MPa (H_2_/CO_2_ (*v*/*v*) = 4:1). Interestingly, the recyclability and stability of the encapsulated composites were much better than those of homogeneous ones, where the activity of [Ru]@UiO-66 was maintained after five successive cycles. The homogeneous composite lost more than half of its original activity in the second cycle, which was likely due to the biomolecular deactivation [48].

**Figure 4:**
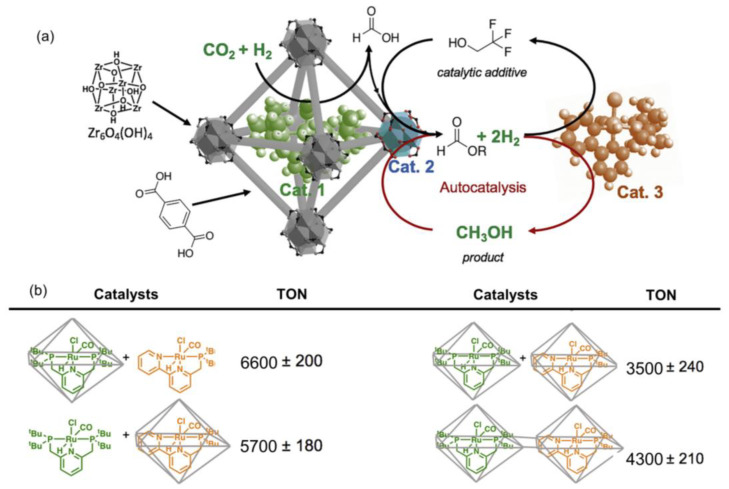
(**a**) The scheme of cascade hydrogenation of CO_2_ to CH_3_OH on MOF–molecular complex host–guest composites. (**b**) The turnover number (TON) was summarized over various kinds of hybrid composites. Reproduced with permission [51]. Copyright 2020, Elsevier Inc. 2020.

Apart from formic acid as the main product during CO_2_ hydrogenation, CH_3_OH also could be produced on MOF-confined molecular complexes. Inspired by biological organisms [76,77], Rayder et al. developed a multi-component catalyst system by integrating [Ru]@MOF and another Ru molecular catalyst to achieve a three-step cascade CO_2_ hydrogenation toward CH_3_OH. More specifically, the molecular complexes (tBuPNP)Ru(CO)-HCl (Ru-1) were firstly encapsulated within the pores of UiO-66 (named Ru-1@UiO-66) by the aperture-opening strategy, where Ru-1 served as an active site to hydrogenate CO_2_ to formic acid [71], and the zirconium oxide nodes with Lewis acidity in UiO-66 served as active sites to catalyze formic acid to a formate ester [78]. Then, another homogenous Ru-based complex (tBuPNN)RuH(CO)Cl (Ru-2) was necessary to catalyze ester hydrogenation to CH_3_OH, as illustrated in Figure 4a [79]. This multi-component catalyst system could deliver a TON of 4710 ± 150 in DMF in the presence of ethanol at 70 °C and 4 MPa (H_2_/CO_2_ (*v*/*v*) = 37:3) after 16 h. In contrast, Ru-1, UiO-66, or Ru-2 ([Ru-1, Ru-2]@UiO-66) were found to be inactive, when independently or physically mixed for CO_2_ hydrogenation. More interestingly, the co-encapsulation of Ru-1 and Ru-2 in UiO-66 was also prepared successfully, which exhibited higher activity toward CH_3_OH than that of mixtures of Ru-1@UiO-66 and Ru-2@UiO-66 as shown in Figure 4b. Even though some activity loss was observed over [Ru-1, Ru-2]@UiO-66, the durability was enhanced and no significant loss was found during the five successive cycles, which was likely due to the isolated sites achieved by the MOF avoiding possible bimolecular decomposition pathways [51]. In this study, UiO-66 not only served as the host material, but also provided a solid Lewis site to transform formic acid to formate ester.

**Figure 5:**
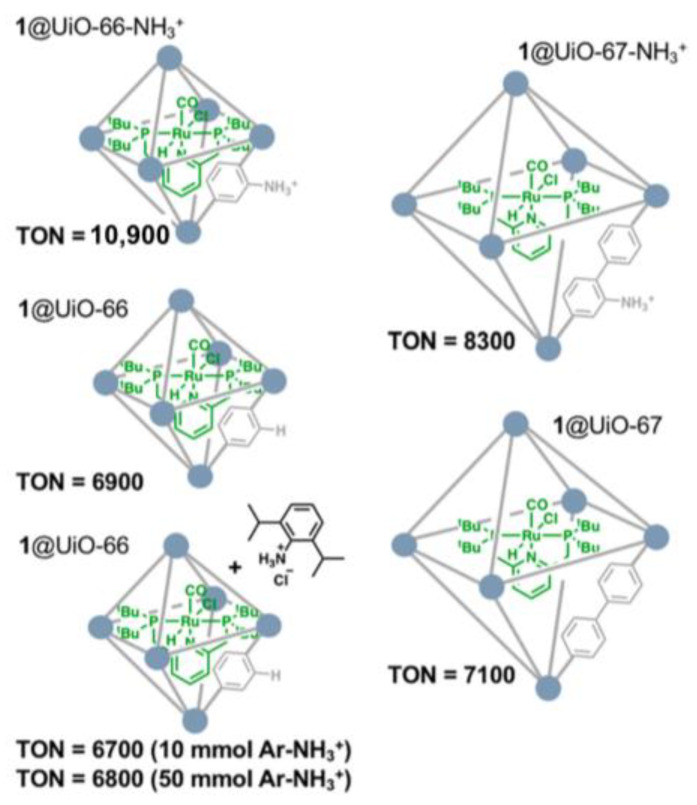
Effect of the pore size of the MOF cage (UiO-66 and UiO-67) and external anilinium functional groups on turnover number (TON) for CO_2_ hydrogenation to CH_3_OH at 70 °C and 4 MPa (H_2_/CO_2_ (*v*/*v*) = 37:3). Reproduced with permission [52]. Copyright 2018, American Chemical Society.

Beyond the primary coordination sphere, second-sphere interactions in host–guest multicomponent catalysts also significantly affect the catalytic activity and selectivity [80,81,82,83,84,85,86]. Tsung and coworkers extensively investigated the second sphere interactions in multicomponent catalyst systems by combining ligand design in the MOFs and the aperture-opening encapsulation strategy for CO_2_ hydrogenation to CH_3_OH [52]. The structure–activity relationships during CO_2_ hydrogenation were efficiently established using various functionalized UiO-66-X hosts (X = -CH_3_, -F, -Br, -NO_2_, -NH_2_, and -NH_3_^+^), as shown in Figure 5, where the UiO-66-NH_3_^+^ host was found to significantly increase CH_3_OH activity compared with that utilized by other UiO-66-Xs as the host. Mechanistic experiments revealed that the NH_3_^+^ functionality could serve as Brønsted acid and facilitate the CO_2_ hydrogenation to formic acid with autocatalytic features. Importantly, the synergistic effect from the host worked only when the functional group was physically close to the encapsulated molecular complexes. The combination of Ru-1@UiO-66-NH_3_^+^ and Ru-2@UiO-66 could achieve the highest TON of 10,900 at 70 °C and 4 MPa (H_2_/CO_2_ (*v*:*v*) = 37:3) in molecular sieves treated with DMF with 10^−5^ mmol 2,2,2-trifluoroethanol after 16 h [52].

**Figure 6:**
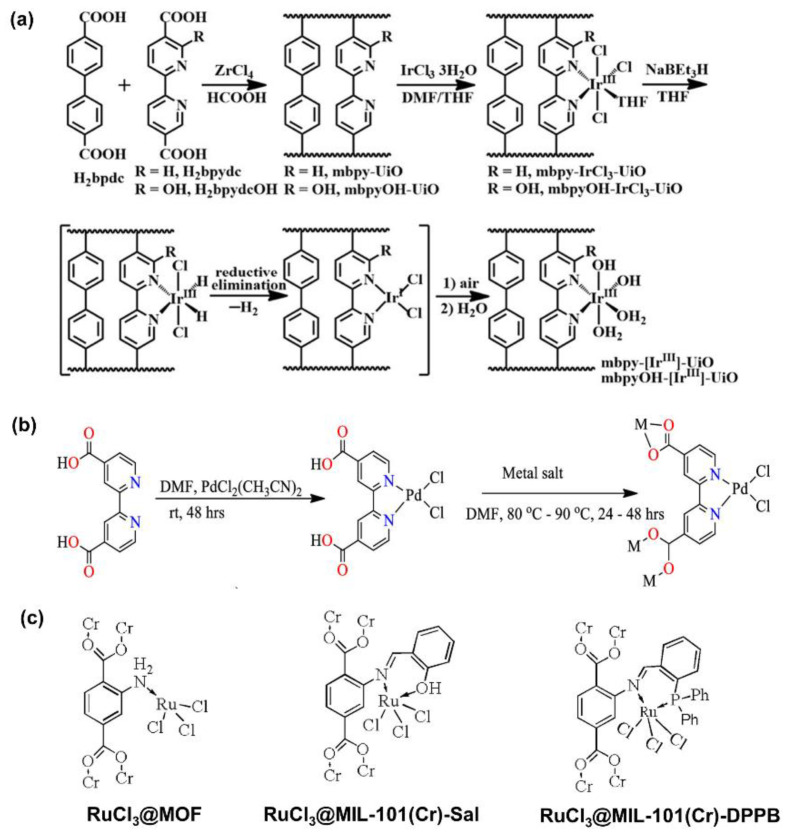
(**a**) The scheme of the host–guest composites (mbpyOH-[Ir^III^]-UiO and mbpy-[Ir^III^]-UiO) preparation. Reproduced with permission [94]. Copyright 2017, American Chemical Society. (**b**) The scheme of the host–guest composite (Pd@Mg:JMS-2). Reproduced with permission [95]. Copyright 2020, American Chemical Society. (**c**) The molecular structure of the as-prepared Ru-based catalysts. Reproduced with permission [96]. Copyright 2019, Elsevier.

Despite the above advances, it still remains challenging to realize the precise control of the position of molecular complexes in MOFs [52,87,88,89,90]. To achieve the controllable modulation of the local environment of active sites in MOFs, organic linkers with some functional groups have been introduced to anchor the metal ions [75,91,92], mimicking ligands in organometallic complexes to stabilize homogeneous organometallic complex catalysts in a solid matrix [93]. An et al. reported a novel heterogenized molecular catalyst for CO_2_ hydrogenation to formic acid via incorporating Ir^III^ ions into UiO-type MOFs using post-synthetic metalation followed by NaBhEt_3_ treatment, where 2,2′-bipyridine-5,5′-dicarboxylate ligands (bpydc) with or without −OH substitution of the 6-position were synthesized, as shown in Figure 6a. The TON of as-obtained bpydcOH-Ir^III^-UiO and bpydc-Ir^III^-UiO achieved 6149 and 417 at 85 °C and 0.1 MPa (H_2_/CO_2_ (*v*:*v*) = 1:1) over 15 h, respectively. The isotopic effect and DFT calculations revealed that concerted proton–hydride transfer was the RDS of CO_2_ hydrogenation. Then, the electron-donating groups from -OH and the pyridyl nitrogen were ascribed to facilitate the RDS [94]. Tshuma et al. presented the rational design and synthesis of novel isostructural MOFs containing catalytically active Pd(II) sites by using 2,2′-bipyridine-4,4′-dicarboxylate linkers, named (Mg(bpdc)(DMF)_2_PdCl_2_]n (Pd@Mg:JMS-2) and [Mn(bpdc)(DMF)_2_PdCl_2_]n (Pd@Mn:JMS-2)), respectively. Figure 6b shows the packing diagram and channel structure of Pd@Mg:JMS-2. For CO_2_ hydrogenation to formic acid in ethanol with KOH as the base at 100 °C and 5 MPa (H_2_/CO_2_ (*v*:*v*) = 4:1), Pd@Mn:JMS-2 and Pd@Mg:JMS-2 gave the TOF values of 409 h^−1^ and 303 h^−1^, respectively, which were higher than that of the homogeneous complex of 170 h^−1^ (Pd directly anchored on 2,2′-bipyridine-4,4′-dicarboxylate). The presence of open metal sites and the encapsulation effect are beneficial to concentrate the CO_2_ and H_2_ gases and avoid deactivation. The reaction mechanism was proposed as follows: (1) H_2_ was first activated to form Pd-dihydride intermediate; (2) the Pd-hydride complex attacked CO_2_ to generate the formate complex, then desorbed to regenerate the active sites [95].

It was also noticed that different linkers could significantly affect the catalytic performance during CO_2_ hydrogenation. Wang et al. successfully immobilized Ru-based molecular complexes into terminal amino functionalized MIL-101(Cr)-NH_2_ by post-synthetic modification, where salicylaldehyde (Sal) and 2-diphenylphosphinobenzaldehyde (DPPBde) were utilized as bridging linkers between MOF and Ru (III) ions. The structure of active sites is shown in Figure 6c, where RuCl_3_ coordinated with the amino group in the MIL-101 (Cr)-NH_2_ (RuCl_3_@MOF), the N atom of carbon–nitrogen double bonds and the O atom of Sal (RuCl_3_@MIL-101(Cr)-Sal), and the N atom of carbon–nitrogen double bonds and the P atom from linker DPPBde (RuCl_3_@MIL-101(Cr)-DPPB), respectively. Among them, RuCl_3_@MIL-101(Cr)-DPPB delivered the highest TON of 242 under 120 °C and 6 MPa (H_2_/CO_2_ (*v*:*v*) = 4:1) with triethylamine (Et_3_N) as the base in ethanol during CO_2_ hydrogenation toward formic acid. Mechanistic insights suggested that the stronger electron-donating ability of P sites in DPPBde promoted the RDS, namely, the insertion of CO_2_ into Ru-H [96]. Similarly, ZIF-8 was also utilized as the host, and 3-methyl-1,2,4-triazole with uncoordinated N sites was introduced to anchor Ru by post-synthetic modification, which was examined for CO_2_ hydrogenation to formic acid. The donated electron to Ru from uncoordinated N sites in linkers decreased the activation energy of CO_2_ activation, as revealed by experiments and theoretical calculations [97].

Overall, the host–guest composites not only maintain the distinctive activity in low-temperature CO_2_ hydrogenation and increase the stability and recyclability of molecular complexes but also provide a platform to further enhance their activity and/or selectivity via engineering the primary coordination sphere and second sphere interactions. The one-pot synthesis is relatively simple to encapsulate molecular complexes into an MOF cage or channel, but it is limited by the size of the MOF channel and remains challenging to tune the encapsulation location and uniformity. It is worth noting that guest molecule leaching always hinders this kind of catalyst in large-scale applications. The post-synthetic modification via the aperture-opening strategy could break the limitation of the MOF channel and guest molecule leaching, but the underlying mechanism remains elusive, impeding the rational design. Moreover, these strategies are applicable only after sophisticated synthetic techniques to construct the desired structure, which would limit large-scale applications. Thus, the development of novel and efficient strategies to prepare MOF-based host–guest composites is highly desired.

### 2.2. Active NPs Confined into MOFs’ Cavities or Channels

Nanometals or oxides have been demonstrated to be effective catalysts for CO_2_ hydrogenation [3,98,99]. Nanoscale effects have been well examined in the rational design of high-performance catalysts due to their specific structural and electronic effects (e.g., facet, coordination, or unsaturated sites) [58,100,101]. High surface-area supports (e.g., zeolite, carbon, oxide, and MOF) are needed to stabilize active sites due to their thermodynamic instability at the nano-/atomic scale, especially at high temperature and pressure conditions in the presence of H_2_O. Among them, the active sites confined in MOFs provide more opportunities, resulting from their chemically tunable pore surfaces [102], framework flexibility [103], and various modification strategies (e.g., post-synthetic modification and exchangeable ligands) [104,105]. Apart from active NPs directly confined in MOFs, the functionalization of linkers with catalytically active species [93,106] and utilization of the modified MOF nodes as the active sites [107] were also designed. The functionalized linkers with different chemical groups could also enhance reactant adsorption [108,109], promoting CO_2_ hydrogenation. In this section, the strategies for MOFs confined in NPs catalysts will be reviewed and the confinement effects will be presented, including size effects, encapsulation effects, and synergistic effects, to provide deeper insights into the advantages and features of confined catalysis.

#### 2.2.1. Atomically Active Sites Confined in MOFs

The electronic and geometric structures of supported NPs can result in dramatic changes in catalytic performance [110,111,112,113,114]. Atomically active sites have been well studied for hydrogenation reactions due to their unique electronic and geometric structures, 100% atom utilization, and unique reaction micro-environments [115,116,117,118]. Recent theoretical and experimental studies have found that atomically active sites exhibit better catalytic activity or selectivity than their nanometer-sized counterparts [118,119,120]. Moreover, the rapid development of related synthetic strategies, characterizations, and theoretical interpretation of atomically active sites provide an ideal platform to understand the structure–performance relationship and reaction mechanisms.

**Figure 7:**
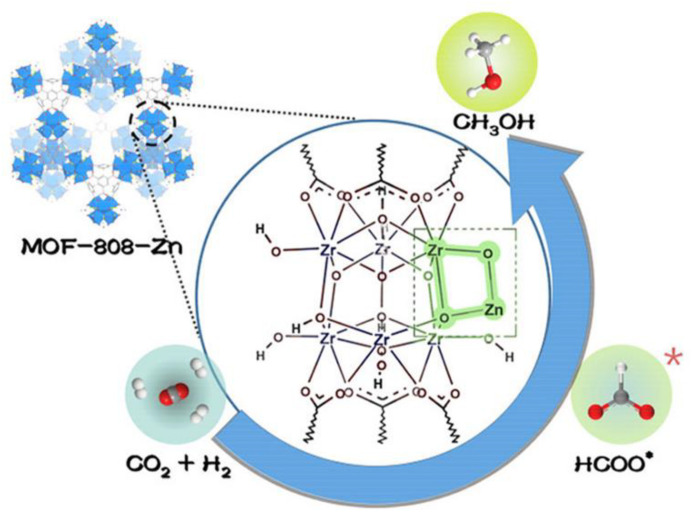
The scheme of neighboring Zn–Zr sites in a metal–organic framework for CO_2_ hydrogenation and HCOO is the important intermediate. Reproduced with permission [121]. Copyright 2021, American Chemical Society.

A unique example is that a well-defined MOF containing functionalized linkers with catalytically active species was utilized as a model material to interpret the reaction mechanism during CO_2_ hydrogenation to examine the structural requirements for CH_3_OH production on ZrZnO_x_. The well-defined Zn^2+^–O–Zr^4+^ sites were constructed by post-synthetic treatment of Zr_6_(μ_3_-O)_4_(μ_3_-OH)_4_ nodes of MOF-808 with ZnEt_2_ and then followed by a mild thermal treatment to remove capping formats on Zr SBUs. The as-prepared composites delivered a high CH_3_OH yield of 5.72 mmol_CH3OH_ g_Zn_^−1^ h^−1^, >99% CH_3_OH selectivity, and excellent stability (e.g., >100 h) in CO_2_ hydrogenation at 250 °C. A synergistic effect between open Zr^4+^ sites and Zn^2+^ centers was proven to be indispensable to the generation of CH_3_OH. Mechanistic investigations disclosed that (1) Zn^2+^ was responsible for H_2_ activation by TPD of H_2_ and H/D exchange tests; (2) CO_2_ was adsorbed and activated on open Zr^4+^ sites in the nearby Zr^4+^–O–Zn^2+^ interface, as shown in Figure 7. In situ DRIFT and DFT calculations further showed that the activated CO_2_ reacted with the heterolytic splitting H^−^ to form *HCOO intermediate on Zn^2+^ sites, followed by hydrogenation to dioxomethylene (*H_2_COO), formaldehyde (*H_2_CO), methoxy (*H_3_CO), and finally methanol [121]. These findings demonstrated that Zn was active for H_2_ activation; meanwhile, both Zn and Zr participated in CO_2_ activation, in agreement with previous studies [10,31,122]. Moreover, this study revealed the precise chemical structures of Zn and Zr in their active forms during CO_2_ hydrogenation.

**Figure 8:**
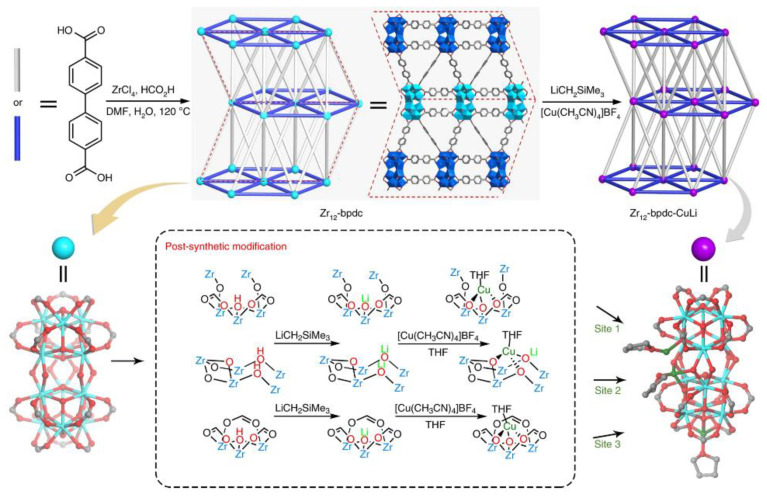
Synthesis and structures of Zr_12_-bpdc and Zr_12_-bpdc-CuLi catalysts. Reproduced with permission [123]. Copyright 2019, Nature publishing group.

Multifunctional sites in MOFs were also facilitated, enabling cascade CO_2_ hydrogenation to multi-carbon products (e.g., C_2_H_5_OH and C_2_H_4_). An et al. designed well-defined Cu active sites on deprotonated [Zr_12_O_8_(μ_3_-O)_8_(μ_2_-O)_6_(carboxylate)_18_]_14_^−^ SBUs in the Zr_12_-MOF for selective CO_2_ hydrogenation to ethanol [123]. Regarding the synthesis, Zr_12_-SBUs with an average thickness of 50 nm were prepared and the SBUs were deprotonated by LiCH_2_SiMe_3_, followed by metalation with [Cu^I^(CH_3_CN)_4_](BF_4_), which was confirmed by infrared (IR) spectrum. Two types of Cu species were formed: 1) four-coordinated tetrahedral Cu^I^ centers([(μ_4_-O^−^)(μ_2_OCO carboxylate)_2_ Cu^I^(THF)]) (site 1, 3) and [(μ_4_-O−Li^+^)(μ_3_-O−)(μ_4_-O−)Cu^I^(THF)] (site 2), as shown in Figure 8. Moreover, different alkali cations were also introduced into the MOF structure to modify the local environment of Cu, which was confirmed by XAS analysis. Among them, the Cs^+^-modified MOF catalyst achieved a TON of 4080 and >99% selectivity of ethanol in supercritical CO_2_ (30 MPa CO_2_ /5 MPa H_2_) at 85 °C. In contrast, the randomly supporting active Cu sites on ZrO_2_ preferred CH_3_OH formation under identical reaction conditions, as the aggregation occurred. The experimental results and DFT calculations disclosed that the H_2_ was activated on (Cu^I^)_2_ sites by a bimetallic oxidative addition process to form (Cu^2+^–H^−^)_2_ and then reacted with CO_2_ to produce methanol and *CHO species, where electron-rich Cu^I^ induced by the effective electron-donating effect of Cs could facilitate these steps. The C–C bond was formed to get the CH_3_CHO intermediate via the nucleophilic attack on methanol by the CHO intermediate on bimetallic Cu sites; ethanol was formed by further hydrogenation. Additionally, alkali metals could stabilize *CHO intermediate to direct the C_2_H_5_OH formation. Overall, the synergistic effect between the cooperative nature of the bimetallic Cu^I^_2_ centers and the electron-rich environment for the Cu center was interpreted to contribute to the higher activity for C_2_H_5_OH formation.

**Figure 9:**
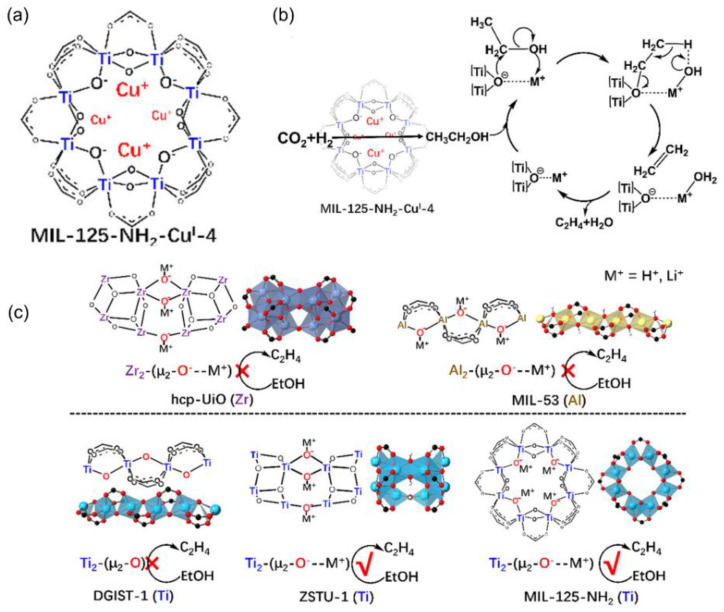
Multiple Cu^+^ supported on a MIL-125 catalyze CO_2_ hydrogenation to C_2_H_4_. (**a**) The local structure of catalyst. (**b**) The scheme of the tandem reaction and the ethanol dehydration mechanism. (**c**) Scheme of the C_2_H_5_OH conversion capability of different MOFs and their derivatives. Reproduced with permission [126]. Copyright2021, American Chemical Society.

To better understand the multi-functionality of MOFs, MOFs involved in catalytic cycles were also investigated. For example, Zeng et al. developed a cascade catalyst, Cu^I^ centers supported on MIL-125 ([Ti_8_(μ_2_-O)_8_(μ_2_-OH)_4_(BDC)_6_]), where the Cu^I^ catalyzed CO_2_ hydrogenation to C_2_H_5_OH, but C_2_H_5_OH dehydration on Ti_2_-μ_2_-O–M^+^ (M^+^ = H^+^, Li^+^) sites occurred. The post-synthetic metalation of SBUs was utilized for catalyst preparation and MIL-125-NH_2_-Cu^I^-4 was obtained, as shown in Figure 9a [124,125]. The as-obtained composites exhibited >90% selectivity toward C_2_H_4_ during CO_2_ hydrogenation with a reaction rate of up to 514 μmol g_cat_^–1^ h^–1^ at 100 °C and 5 MPa (H_2_/CO_2_ = 3:1) in THF. The choice of MIL-125 as the support was critical to the tandem CO_2_-to-C_2_H_4_ transformation: (1) the Ti_8_(μ_2_-O)_8_(μ_2_-OH)_4_ SBU rather than other SBUs stabilized Cu^I^ to avoid Cu^0^ formation; (2) the short Cu^I^–Cu^I^ distance in Ti_8_(μ_2_-O)_8_(μ_2_-O-CuI)_4_ enhanced the synergistic effect on C_2_ formation; (3) the strong Lewis acid Ti^IV^ catalyzed C_2_H_5_OH dehydration at low temperatures (∼100 °C); and (4) C_2_H_5_OH was easily adsorbed on the Ti_2_(μ_2_-O–M^+^) site in Ti_8_(μ_2_-O)_8_(μ_2_-O–CuI)_4_, which further induced β-elimination to produce C_2_H_4_ (Figure 9b,c). This work highlighted new opportunities in using MOFs as novel supports for CO_2_ hydrogenation to C_2_H_4_ [126]. Moreover, this study indicated the pore-dependent activity and selectivity during CO_2_ hydrogenation in tunable and ordered structure MOFs.

**Figure 10:**
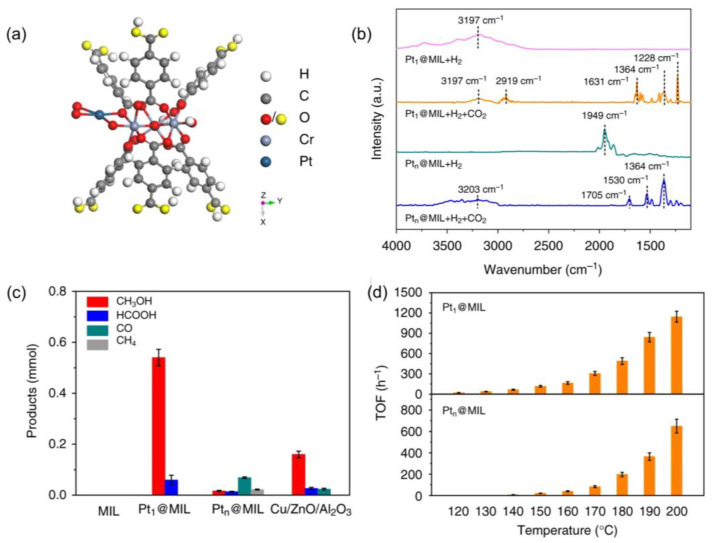
(**a**) Structural model of Pt_1_@MIL. (**b**) The in situ DRIFT of CO_2_ hydrogenation over Pt_1_@MIL. (**c**,**d**) The comparison of catalytic performance over various catalysts during CO_2_ hydrogenation. Reproduced with permission [127]. Copyright 2019, Nature publishing group.

Apart from the transition metals (Zn or Cu), noble metals (e.g., Pt) are also able to encapsulate into MOFs, where the atomically dispersed Pt_1_ atom was coordinated by four O atoms in MIL-101, as shown in Figure 10a. The active sites created by metal–ligand cooperativity led to the dissociation of H_2_ to form hydroxyl groups, attacking CO_2_ to produce the key intermediates of HCOO*, evidenced by DFT calculations and operando spectroscopies (Figure 10b). In contrast, the Pt cluster (Pt_n_) encapsulated in MIL-101 preferred hydride formation and then hydrogenated CO_2_ into COOH* as key intermediates. The divergence in reaction paths resulted in different catalytic selectivity between Pt_1_@MIL-101(90%) and Pt_n_@MIL-101 (58%), where the *HCOO intermediates went through stepwise hydrogenation to produce CH_3_OH, while *COOH was hydrogenated into various products including HCOOH, CH_3_OH, CO, and CH_4_ (Figure 10c,d) [127]. 

Accordingly, atomically dispersed active sites in MOFs are an ideal target in catalyst design, especially for noble metals, which not only increase the exposed active sites per molar amount of metals but also create new prospects for the controllable surface free energy of the metal atoms. The synergistic effect in MOFs is of great importance for the rational design of catalysts to achieve excellent performance for desired products. The design of atomically distributed active metals in MOFs with reasonable catalytic performance has been demonstrated in the lab; however, no related application was found on a large scale, hence, more efforts should be directed to developing more efficient strategies for confined catalysts using MOFs as hosts.

#### 2.2.2. Active NPs Confined in Monocrystal MOF

Heterogeneous catalysts are usually prepared by loading active metal sites onto a support, where both the structure of active metal sites (e.g., facet, morphology, and electronic state) and properties of supports (physical properties and chemical properties) will affect the catalytic performance [116,128,129,130,131,132,133]. For example, the different facets of active NPs result in different unsaturated metal surface atoms, which can change the catalytic processes; the supports not only affect the dispersion of active NPs but also tune the electronic and/or geometrical structure of active NPs via SMSI [132,134,135,136]. Actually, the sintering under harsh reaction conditions due to Ostwald ripening or particle migration and coalescence makes it challenging for industrial application [137,138]. An efficient and sustainable strategy, encapsulation has been well investigated to increase their long-term stability [139]. 

**Figure 11:**
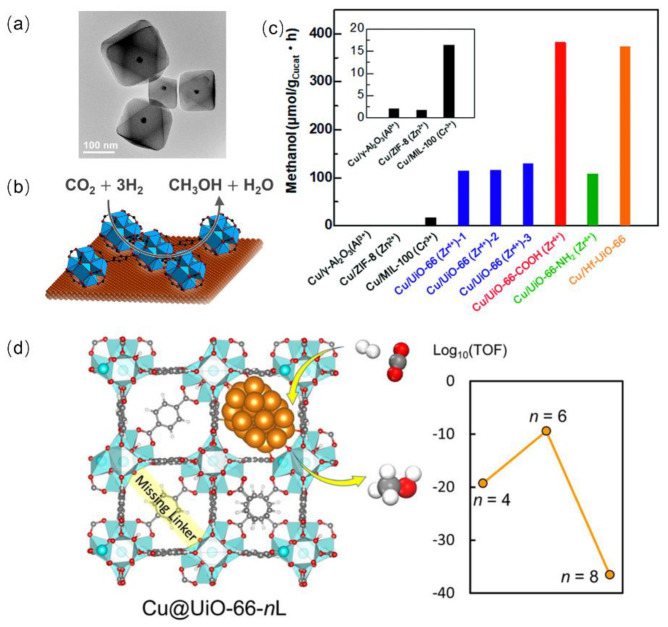
(**a**) TEM image of Cu@UiO-66. (**b**) The scheme of CO_2_ hydrogenation over Cu@UiO-66. Reproduced with permission [140]. Copyright 2016, American Chemical Society. (**c**) The amount of CH_3_OH synthesized from CO_2_ and H_2_ using Cu/γ-Al_2_O_3_ and various Cu/MOF composite catalysts. Reproduced with permission [141]. Copyright 2019, Royal Society Chemistry. (**d**) The structure–performance relationship of Cu@UiO-66 during CO_2_ hydrogenation. Reproduced with permission [143]. Copyright 2022, American Chemical Society.

Rungtaweevoranit et al. successfully synthesized single nanocrystalline UiO-66 encapsulated 18 nm Cu via the bottom-up method using Zr(OPr^n^)_4_ as a precursor (Figure 11a,b). The as-prepared Cu@UiO-66 was demonstrated to be active and selective for CO_2_ hydrogenation toward CH_3_OH, and the TOF was 3.7 × 10^−3^ s^−1^ at 175 °C and 1 MPa (H_2_/CO_2_ = 3/1). The formation rate of CH_3_OH on Cu@UiO-66 was almost two and nine times higher than those of Cu supported on UiO-66 and commercial Cu/Zn/Al_2_O_3_, respectively. Moreover, 100% selectivity was observed on Cu@UiO-66. The strong interaction between Cu NPs and Zr oxide SBUs of the MOF was interpreted as leading to the higher catalytic performance [140]. Kobayashi et al. investigated the coated effects of MOFs including ZIF-8, MIL-100, and functionalized UiO-66 on CO_2_ hydrogenation for CH_3_OH formation, as shown in Figure 11c. The charge transfer was observed between Cu and MOF substrates, and UiO-66 was found to be the most active support. Interestingly, the replacement of Zr^4+^ with Hf^4+^ or functional groups (e.g., from -NH_2_ to -COOH) in UiO-66 enhanced the rate of CH_3_OH production [141]. Truhlar and coworkers provided deeper insight into the molecular interactions between Cu and Zr in UiO-66. The experimental and theoretical results disclosed that the direct interaction between Cu NPs and ZrO_2_ nodes was essential for CO_2_ hydrogenation to CH_3_OH (Figure 11d) [142]. The quantitative effects of the missing-linker defects on H_2_ and CO_2_ activation were carried out using a detailed quantum mechanical study. It was found that the optimum number of missing linkers (ca. 5–7 per unit cell) balanced the steric effects and the strong CO_2_ binding on the ZrO_2_ node delivered a maximum TOF of CH_3_OH formation [143].

MOFs further provide a specific platform for NPs including protective microenvironments and functional sites. Zheng et al. reported that the core–shell monodispersed nanosphere Au@Pd could be encapsulated by the UiO-66 membrane to protect its morphology and then the microporous characteristic of UiO-66 preferred the adsorption of Pt NPs on its surface to impart its functionality. This kind of assembly enhanced the interaction between NPs and UiO-66, allowing for the spatial distribution of NPs in MOFs. Furthermore, the catalysts for the RWGS in a fixed-bed flow reactor exhibited high catalytic activity and CO selectivity [144].

**Figure 12:**
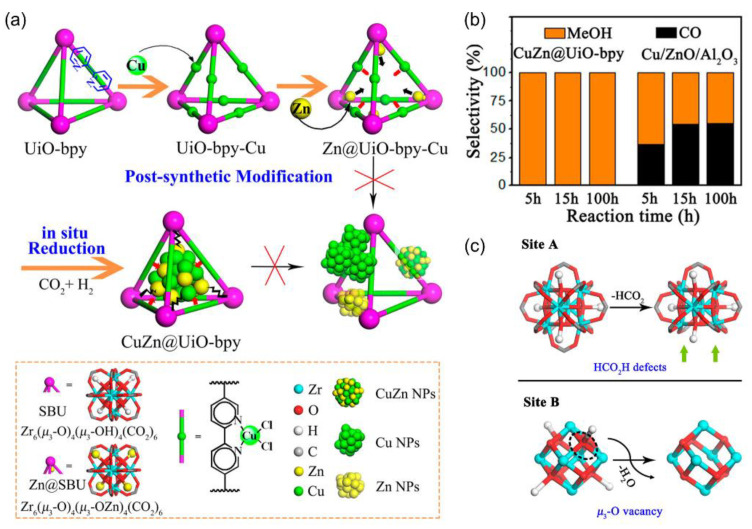
(**a**) The scheme of preparing CuZn@UiO-bpy via in situ reduction of post-synthetically metalated UiO-bpy. (**b**) Selectivity of CH_3_OH vs reaction time over CuZn@UiO-bpy and Cu/ZnO/Al_2_O_3_. (**c**) Formation of unsaturated Zr sites that can accept CO_2_ and hydrogen spillover from Cu surfaces. Reproduced with permission [145]. Copyright 2017, American Chemical Society.

MOFs can also serve as novel supports for mixed oxide catalysts via taking advantage of tunable and specific strong metal–support interactions. Lin and coworkers demonstrated a strategy to utilize the UiO-bpy MOF constructed by bpy and Zr_6_(μ_3_-O)_4_(μ_3_-OH)_4_ to encapsulate ultrasmall Cu/ZnO_x_ nanoparticles, which selectively catalyzed high-rate CO_2_ hydrogenation to CH_3_OH with the yield up to 2.59 g_CH3OH_ kg_Cu_^–1^ h^–1^, 100% CH_3_OH selectivity, and high stability over 100 h (Figure 12a). The UiO-bpy MOF was chosen because of its exceptional hydrothermal stability. Post-synthetic metalation was developed to introduce Cu^2+^ and Zn^2+^ ions sequentially, which were coordinated to the bpy and μ_3_-OH sites in the MOF, respectively. Finally, ultra-small Cu/ZnO_x_ NPs generated under reaction conditions were encapsulated in the tetrahedral and octahedral cages confined by the ligands as the active sites. The as-prepared composite exhibited much better CH_3_OH selectivity and stability than those of commercial Cu/ZnO/Al_2_O_3_ (Figure 12b), likely resulting from the suppression of Cu NPs’ agglomeration and phase separation between Cu and ZnO_x_. Regarding mechanistic understanding, the authors proposed that CO_2_ was adsorbed on unsaturated ZrO_x_ and ZnO_x_ sites to form carbonates and bicarbonates; the homolytic dissociation of H on Cu spill over to the Zr sites on the SBUs and defect sites of ZnO_x_ (Figure 12c). Overall, the synergistic combination of hydrogen activation sites on Cu and CO_2_ activation on ZnO_x_ and Zr SBUs contributes to superior performance of the CuZn@UiO-bpy catalyst [145]. Similarly, Yu et al. also reported that ultra-small bimetallic Cu/ZnO_x_ NPs was encapsulated in UiO-66 by the deposition–precipitation method; both metal loading and the Cu/Zn mole ratio could regulate the Cu-Zn interaction. The highest CH_3_OH yield of 9.1% was obtained over the Cu-Zn@UiO-66 catalyst (Cu-Zn loading: 35 wt.% and Cu/Zn mole ratio: 2.5) [146].

#### 2.2.3. Active Species Confined in MOF Membrane

**Figure 13:**
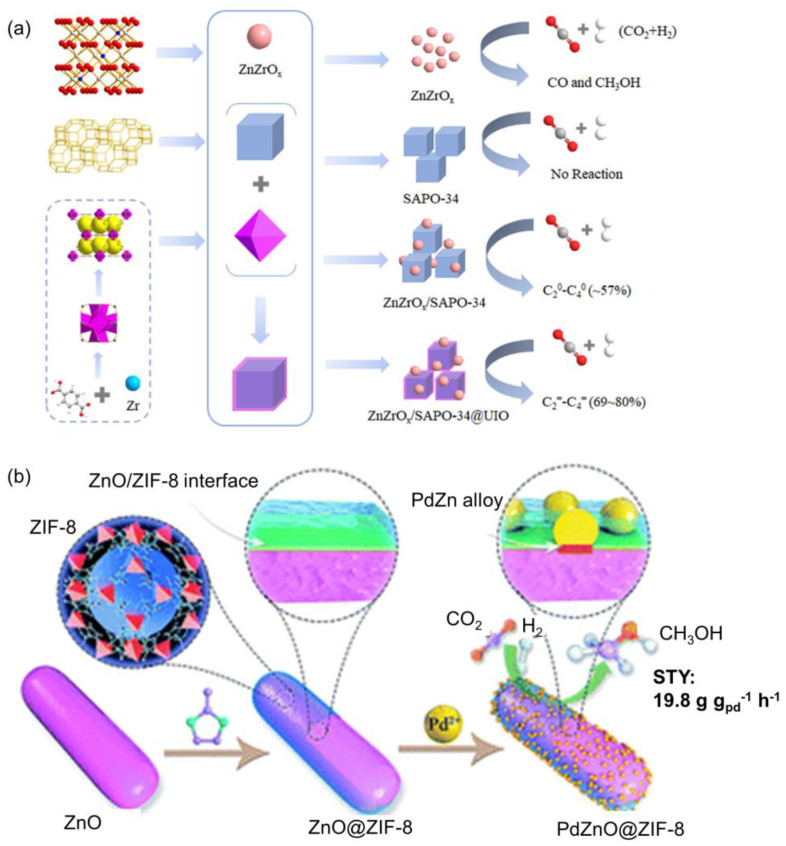
(**a**) The scheme of the ZnZrO_x_/SAPO-34@MOF catalyst assembly for CO_2_ hydrogenation. Reproduced with permission [147]. Copyright 2022, American Chemical Society. (**b**) Confinement of PdZn alloy in a defect-enriched ZnO/ZIF-8 interface for efficient and selective CO_2_ hydrogenation to CH_3_OH. Reproduced with permission [1]. Copyright 2019, Royal Society of Chemistry.

The active species of CO_2_ hydrogenation are not only encapsulated in monocrystal MOFs, but also confined by the MOF membrane. The combination of guest species and different types of MOF active sites (e.g., metal nodes, functional organic linkers) makes MOFs promising multifunctional materials for synergistic catalysis [47]. For example, the ZnZrO_x_/SAPO-34 composite was able to selectively catalyze CO_2_ hydrogenation to paraffin. Jiang et al. shifted the product distributions from paraffins to olefins via the introduction of the UiO-66 membrane as a coating layer on the SAPO-34 surface. It was demonstrated that the stable UiO-n membrane passivated the excessive Brønsted acid sites of SAPO-34, suppressing the hydrogenation of olefins to paraffins. Meanwhile, the uniform UiO-66 membrane had no effect on the diffusion step during CO_2_ hydrogenation, where the selectivity of the C_2_−C_4_ olefins was increased from 57% on ZnZrO_x_/SAPO-34 to 80% on that with a UiO-66 membrane under 380 °C, 3 MPa, and GHSV of 6000 h^–1^ (CO_2_/H_2_/Ar = 24:72:4), as shown in Figure 13a [147]. Compared with the existing strategies of adjusting the acidity of zeolites by changing the structure of the zeolite framework (e.g., alkali treatment or increasing calcination temperature), the method of membranization by coating a layer of a functional MOF membrane on the zeolite surface did not affect the framework structure. Moreover, a UiO-66 membrane was epitaxially grown on the surface of nano-SAPO-34 clusters as a support for dispersed Pt for CO_2_ hydrogenation. The MOF membrane not only overcomes the noble metal agglomeration and enhances the molecular sieve synergistic catalysis, but also provides sites for CO_2_ adsorption, significantly enhancing the selectivity and yield [148]. Similarly, Pan et al. developed a strategy for the epitaxial growth of the UiO-66-NH_2_ shell to obtain an MOF-membranized bicomponent core−shell catalyst, HZSM-5@UiO-66-NH_2_/Pd. By coating UiO-66-NH_2_ on the surface of HZSM-5, the highly dispersed and small Pd NPs can be anchored on the HZSM-5@UiO-66-NH_2_ supports owing to the affinity between UiO-66-NH_2_ and Pd NPs. The as-prepared HZSM-5@UiO-66-NH_2_/Pd exhibited high CO selectivity (92.2%) with 17.1% CO_2_ conversion in CO_2_ hydrogenation toward CO at 320 °C [149]. 

Apart from Cu/ZnO catalysts, Pd/ZnO catalysts are also active for CH_3_OH production in CO_2_ hydrogenation [150,151]. Li et al. designed core–shell ZnO@ZIF-8 nanorods with an ultrathin ZIF-8 membrane overcoating to confine Pd NPs at the ZnO/ZIF-8 interface for CO_2_ hydrogenation, as shown in Figure 13b. Surface oxygen defects of ZnO were generated during the formation of ZIF-8 shells, which facilitated the chemisorption of CO_2_ by promoting electron transfer to CO_2_. Sub-nano PdZn alloy was confined at the ZnO/ZIF-8 interface after H_2_ reduction, giving a high CH_3_OH selectivity and long-term stability. The thickness of ZIF-8 significantly dominated the activity of CH_3_OH formation, which was related to the number of PdZn alloy sites at the interface with O-defective ZnO. The optimized catalyst achieved 66–78% of CH_3_OH selectivity and 12.1–19.8 g g_Pd_^−1^ h^−1^ of methanol yield at 250–290 °C and 4.5 MPa, showing much higher values than those of reported Pd-based catalysts under comparable conditions [1].

With the rapid development of synthetic chemistry, various MOF-confined catalysts with different structures and interfaces were successfully constructed via one-pot synthesis and post-synthetic modification, which impressively exhibited unique properties over traditionally supported catalysts. The catalytic performance of various catalysts in CO_2_ hydrogenation is summarized in Table 1. However, they still suffered from big challenges, especially MOF stability in fixed-bed type reactors. Most MOFs were easily decomposed in high-polar solvents or at temperatures higher than 300 °C, especially in the presence of water [152,153,154].

## 3. Confined Synthesis and Confinement Effects for MOF-Derived Materials

MOFs have been explored as potential templates or precursors to prepare highly dispersed NPs via thermal decomposition due to their unique structure and textural properties [57,155,156,157]. Carbon-encapsulated NPs derived from MOFs as emerging catalysts received tremendous research efforts [158,159,160]. The framework of MOFs could protect the active site aggregation to generate highly dispersed NPs during high-temperature pyrolysis. Moreover, the confinement effects induced by the encapsulation carbon layer provide robust microenvironments for CO_2_ hydrogenation, which could tune the binding energy of key intermediates and affect the adsorption of CO_2_ and transportation of products. In this section, we will simply highlight some important merits of the encapsulated metal nanoparticles for CO_2_ hydrogenation, including modification of local reaction environment, electronic transfer, and interfacial catalysis to enhance catalytic performance. 

**Figure 14:**
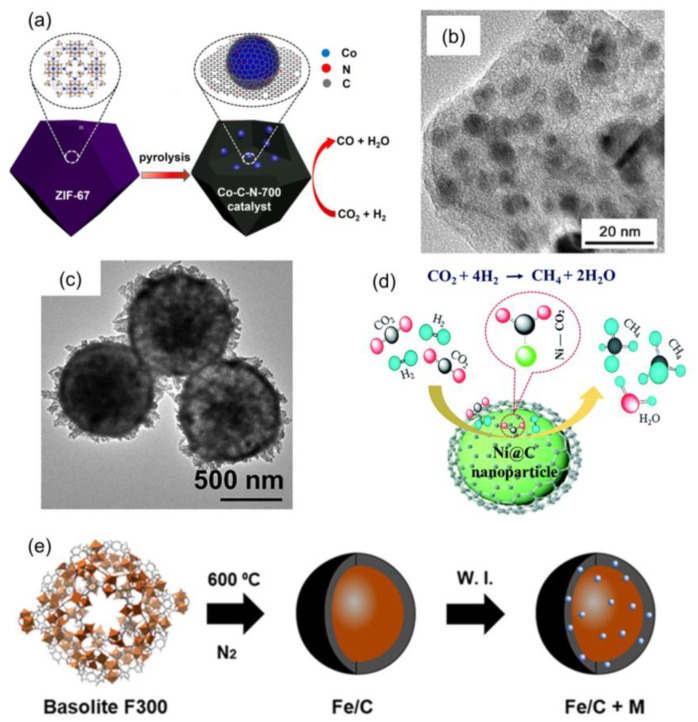
(**a**) The scheme of cobalt-based nonprecious metal catalysts derived from MOFs for selective CO_2_ hydrogenation to CO. (**b**) TEM image of Co-C-N-700 catalyst. Reproduced with permission [161]. Copyright 2019, American Chemical Society. (**c**) TEM image of the hierarchical Ni@C hollow spheres. (**d**) The scheme of Ni-based nonprecious metal catalysts derived from MOFs for selective CO_2_ hydrogenation to CH_4_. Reproduced with permission [168]. Copyright 2019, Royal Society of Chemistry. (**e**) The scheme of the preparation of the Fe-based catalyst. Reproduced with permission [56]. Copyright 2018, American Chemical Society.

Carbon-encapsulated highly dispersed NPs derived from MOFs were widely investigated for selective CO_2_ hydrogenation. Lu et al. reported a one-step pyrolysis of MOFs containing both an N-free and an N-containing linker to obtain highly dispersed cobalt NPs embedded in a carbon matrix for selective CO_2_ hydrogenation to CO (Figure 14a,b) [161]. The presence of N in the MOF precursor could not only decrease the size of generated Co [162,163], but also modulate local reaction conditions because of the formation of a Mott–Schottky interface, which lowered the energy barrier for the formation of formate intermediates [164,165,166]. Similar enhancement was also observed in Fischer–Tropsch synthesis [167]. Moreover, Ni-based MOFs could generate hierarchical Ni@C hollow spheres composed of highly dispersed Ni NPs confined in carbon shells, which was active and selective for CO_2_ hydrogenation to CH_4_ (Figure 14c,d). The high surface area and highly dispersed active sites were ascribed to promote CO_2_ adsorption and redox catalysis [168]. Apart from C_1_ products, highly dispersed Fe-based catalysts derived from Fe-MIL-88B could catalyze CO_2_ actively and selectively to valuable hydrocarbons (C_2+_) [169,170,171,172,173,174]. Ramirez et al. examined the promotion effects of various elements (Cu, Mo, Li, Na, K, Mg, Ca, Zn, Ni, Co, Mn, Fe, Pt, and Rh) for the resulting Fe@C-based composites during CO_2_ hydrogenation, where the Basolite F300(Fe) was used as a template and incipient wetness impregnation was utilized to add the various promotors (Figure 14e) [56]. Among them, only K could increase the activity and enhance C_2_–C_6_ olefin selectivity from 0.7% to 36%.

**Figure 15:**
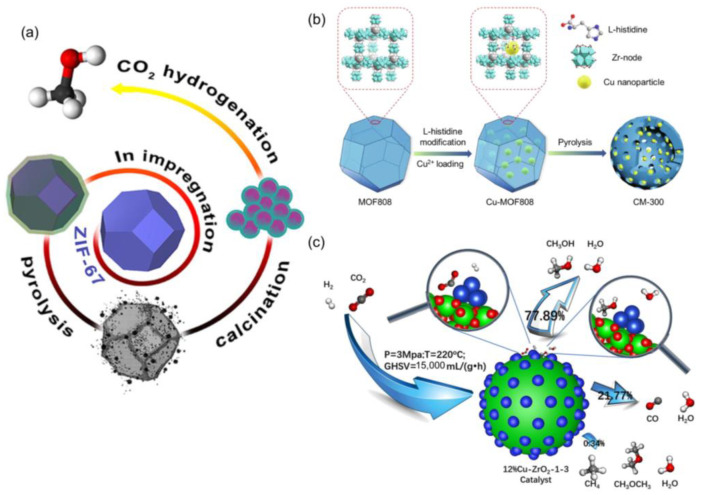
(**a**) The scheme of MOF-derived synthesis of CoIn-based catalysts for CO_2_ hydrogenation. Reproduced with permission [191]. Copyright 2018, American Chemical Society. (**b**) The scheme of catalysts preparation for Cu^+^-ZrO_2_ interfacial sites with highly dispersed copper NPs derived from Cu@UiO-67. Reproduced with permission [197]. Copyright 2018, Elsevier. (**c**) The scheme of highly dispersed Cu NPs on ZrO_2_ derived from ZrO_2_@ HKUST-1 composites for CO_2_ hydrogenation to CH_3_OH. Reproduced with permission [198]. Copyright 2021, Elsevier.

Another property of the confinement effects is the strong interaction between the metal NPs and the encapsulating materials, which facilitates the electron transfer under some conditions and maximizes the interfacial area due to their close contact [175,176,177,178,179,180,181,182,183,184,185,186,187,188,189,190] Pustovarenko et al. demonstrated the MOF-mediated route for the preparation of highly efficient Co_3_O_4_-supported In_2_O_3_ catalyst via stepwise pyrolytic–oxidative decomposition, as shown in Figure 15a, which selectively catalyzed selective CO_2_ hydrogenation to CH_3_OH [191]. The stable Cu-ZnO interfacial sites were also constructed using a novel bimetallic CuZn-BTC MOF for CO_2_ hydrogenation to CH_3_OH [192]. Additionally, Cu-based catalysts were revealed to be structure sensitive, including facet, defect, particle size, and interface [22,193,194,195,196]. Han et al. prepared hollow Cu@ZrO_2_ catalysts through pyrolysis of Cu-loaded Zr-MOF, where low-temperature pyrolysis (e.g., 300 °C) produced highly dispersed Cu nanoparticles with balanced Cu^0^/Cu^+^ sites, larger amounts of surface basic sites, and an abundant Cu-ZrO_2_ interface in the hollow structure, as shown in Figure 15b, which delivered the best performance for CO_2_ hydrogenation to CH_3_OH with 5% CO_2_ conversion and 85% CH_3_OH selectivity at a reaction temperature of 220 °C [197]. Similarly, Yu et al. prepared highly dispersed Cu NPs on ZrO_2_ via the calcination and reduction of ZrO_2_@HKUST-1 (Figure 15c). Due to the confinement of MOFs, highly dispersed Cu on ZrO_2_ was generated, which increased the efficient active sites and produced more interface between the Cu and ZrO_2_. The as-prepared catalysts exhibited 5.2 times higher CH_3_OH yield than that of those catalysts prepared by the conventional impregnation method [198]. Similar enhancement was also demonstrated in the PdZn alloy and In_2_O_3_/Pd during CO_2_ hydrogenation [199,200].

Overall, the MOF-derived materials exhibited higher stability than MOFs themselves in CO_2_ hydrogenation. The electronic effects of SMSI and interfacial catalysis could dramatically enhance catalytic performance. However, the reaction mechanism at the molecular level remains elusive; more advanced operando characterizations in conjunction with theoretical calculation are needed in further work to decipher the mechanism in greater detail. It is worth noting that the encapsulating layer (especially the carbon layer) seems to be consumed on stream to decrease the catalytic performance because of the involvement in CO_2_ hydrogenation.

## 4. Conclusions and Outlook

Different from zeolite with fixed tetrahedral Si/Al coordination and pore sizes (<1 nm) [201], the uniform and tunable cavities and tailorable composite make MOFs especially attractive for heterogeneous catalysis. The confinement effects in MOFs or MOF-derived materials involving immobilization, size of active sites, encapsulation, and synergy effect significantly perturb the catalytic performance of CO_2_ hydrogenation. In this review, the microstructural optimization and engineering of MOF-based catalysts for CO_2_ hydrogenation were systematically summarized. The MOFs or MOF-derived catalysts can be simply divided into three categories: (1) molecular complexes, atomic or nanosized active species are confined into cavities or channels of MOFs via one-pot synthesis or post-synthetic modification; (2) active species are surrounded by MOFs or MOF membranes by epitaxial growth; (3) active sites are embedded in carbon or oxide layers derived from MOFs. The most important merits of the heterogenized molecular complexes are their dramatically enhanced recyclability and stability. The advantages of the confined NPs are the cage or channel stabilization and modification of the local reaction microenvironment. The composites derived from MOFs exhibited strong electronic transfer effects and increased interface catalysis between active sites and the encapsulating layers. 

Despite all the progress achieved by researchers, many problems remain unsolved. For example, the precise synthesis with desired spatial distribution, loading, and size of active species in MOFs represent the biggest challenges in this field; the hydrothermal stability of host MOFs under reaction conditions is still limited; most products are CO, CH_4_, and CH_3_OH, but the high-value-added chemicals or fuels produced from CO_2_ hydrogenation are limited; and the enhancement interpretation of confinement effects at the molecular level is less than well understood. 

In the future, extensive efforts are required to resolve the existing challenges. The detailed directions are as follows:

1. The development of sophisticated organic synthesis to design more linkers with desired functional groups is highly needed, which will make the precise tailoring of active sites highly possible. For example, some functional groups (e.g., carboxyl, sulfonic acid, or amino groups) are effective to stabilize the active sites; therefore, their controllable synthesis with desired positions and contents are essential to obtain rationally designed catalysts.
2. More fundamental interpretations for MOF nucleation, growth, decomposition, and collapsing can provide a strong foundation for more stable MOFs preparations. Since the generated water in CO_2_ hydrogenation is unavoidable, water management near the active sites can change the stability of MOFs and tune product distributions.
3. To date, only imidazole or carboxylate-based MOFs were widely investigated for CO_2_ hydrogenation; more linkers and metal nodes (e.g., Ru, Ti, Mo, and Mn) are needed to obtain a higher diversity of applicable MOFs. Apart from Cu-, Zr-, or Zn-based composites, some new catalyst systems (e.g., Ru, Mn, and Rh) are more interesting for high-value-added chemicals or fuel formation.
4. Advanced characterization techniques are required to reveal the structure of the active sites; the reaction mechanisms at the molecular scale are helpful to increase the selectivity of target products.

## Figures and Tables

**Table 1:** Summary of CO_2_ hydrogenation over various catalysts.

Catalysts	Reaction Conditions			Performance		Ref.
	*P* (MPa)	T (°C)	CO_2_/H_2_	TON	Product	
Ru_3_-NHC-MOF	8	120	1	3803	HCOOH	[65]
[Ru]@UiO-66^1^	1.5	27	4	280,000	HCOOH	[48]
Ru-1@UiO-66+Ru-2	4	70	12	6600	CH_3_OH	[79]
Ru-2@UiO-66+Ru-1				5700		
Ru-1@UiO-66+Ru-2@UiO-66				3500		
[Ru-1, Ru-2]@UiO-66				4300		
Ru-1@UiO-66-NH_3_^+^	4	70	12	10,900	CH_3_OH	[52]
Ru-1@UiO-66				6900		
Ru-1@UiO-67				7100		
Ru-1@UiO-66-NH_3_^+^				8300		
bpydcOH-Ir^III^-UiO	0.1	85	1	6149	CH_3_OH	[87]
bpydc-Ir^III^-UiO				417		
Pd@Mn:JMS-2	5	100	4	409	C_2_H_5_OH	[88]
RuCl_3_@MIL-101(Cr)-DPPB	6	120	4	242	HCOOH	[89]
Zn-MOF-88	4	250	3	5.9 ^a^	CH_3_OH	[121]
[Cu^I^]	35	85	0.2	4080	C_2_H_5_OH	[123]
Cu/MIL-125	5	100	3	514 ^a^	C_2_H_4_	[126]
Pt_1_@MIL-101	3.2	150	3	0.6 ^a^	CH_3_OH (90%)	[127]
CuNP@UiO-66	1	175	3	0.004 ^b^	CH_3_OH	[140]
CuZn@UiO-bpy	4	250	3	2.6 ^c^	CH_3_OH	[145]

Note: [Ru]: (tBuPNP)Ru(CO)HCl; Ru-1: (tBuPNP)Ru(CO)-HCl; Ru-2: (tBuPNN)RuH(CO)Cl; bpydcOH: 2,2′-bipyridine-5,5′-dicarboxylate ligands (bpydc) with −OH substitution on the 6-position; Pd@Mn:JMS-2: [Mn(bpdc)(DMF)2PdCl2]_n_ and 2,2′-bipyridine-4,4′-dicarboxylate (bpdc); DPPB: 2-diphenylphosphinobenzaldehyde (DPPBde); a: mgCH3OH gZn^−1^ h^−1^; [Cu^I^]: [(μ_4_-O−Li^+^)(μ_3_-O−)(μ_4_-O−)Cu^I^(THF)]; b: s^−1^; c: g_MeOH_ kgCu^–1^ h^–1.^

## Data Availability

Not applicable.
